# Supplementary material for: SA-Net: A scale-attention network for medical image segmentation
Source: PLoS One. 2021 Apr 14;16(4):e0247388. doi: 10.1371/journal.pone.0247388 (PMC8046243; doi:10.1371/journal.pone.0247388)
Supplement: Attachment — Submitted filename: Reviewer_plosOne_SANet.pdf [file pone.0247388.s001.pdf]

This paper proposed a scale-attention network upon the backbone network of UNet. And designed multiple attention modulus at different scales, on segmentation tasks. The attention mechanism at scales improved the extraction of global-local features. The proposed method is trained and tested on multiple datasets including two public retina vessel datasets and the LUNA. The method is also validated on the artery/vein classification problem and blastocyst segmentation. The study across multiple datasets are impressive. In addition, the method is compared with extensive baseline methods in Table 1, which is a bonus and provides a good reference for future studies.

Overall, this is a well evaluated paper with multiple applications and large-scale experiments. However, I have several concerns about several places, especially in the description of abstract/introduction and discussion. It would be great to further tune some statements, please see as follows:

1. The abstract address that “uses an attention module to learn and understand which features are the most important for medical image segmentation”. However, this paper didn’t evaluate the feature importance or have the conclusion on which features are the most important. I would recommend the authors to add the evaluation of different features effects or to rephrase the sentence.
2. I suggest changing the description of “not only a waste of manpower and time but also prone” in the first sentence of introduction to “manual effort is time-consuming and tedious”, we should be humble to previous manual work by radiologists and clinicians, these are hard tasks and defines many critical medical problems.
3. “In particular, U-Net [2] achieved the best segmentation accuracy for neuronal structures in electron microscopy.” This claim lacks a citation, could the authors add the citation on the best performed paper of “neuronal structures in electron microscopy”?\
4. Suggestion to reduce redundancy: “and its performance does not deteriorate even when large enough datasets are lacking” to “and its performance does not deteriorate at low data regime”
5. The fourth paragraph in Introduction, “Nevertheless, …., these variants still rely on cascaded multi-stage CNNs. Therefore, we emphasize the design of particular good multi-scale features”. This sentence is confusing, I believe the propose method is still based on encoder-decoder architecture and used the cascaded multi-stage CNNs. Could the authors rephrase the sentence and highlight the difference between the proposed work and convention UNet?
6. The legend of Figure 1 and Figure 2 are too small to see, could enlarge the legend.
7. The experiment setting separated the datasets into training and testing or cross-validation, since there is no validation set, how the final model is selected for testing?
8. Followed by the first concern, the authors highlighted the ability to “better learn and understand the features at different scales” at the Discussion and Conclusion, it would be more intuitive to visualize the attention maps at different scales for reader, and discuss the difference with non-multi-scale attention. Otherwise, I would suggest removing this statement.

Summary:

This is a well-evaluated paper. The large-scale experiments and comparisons are impressive. The application on five different datasets is a bonus. Overall, this is a great study, it should be of potential interest of readers if authors could address above issues and further fine-tune some descriptions.
