# Supplementary material for: SA-Net: A scale-attention network for medical image segmentation
Source: PLoS One. 2021 Apr 14;16(4):e0247388. doi: 10.1371/journal.pone.0247388 (PMC8046243; doi:10.1371/journal.pone.0247388)
Supplement: Attachment — Submitted filename: Response to Reviewers.docx [file pone.0247388.s003.docx]

**Responses for comments**

Dear Editors and Reviewers:

We thank you very much for giving us an opportunity to revise our manuscript again. Thanks a lot for the helpful comments and recommends and thank you for your time spent. According to the reviewers' comments, we have revised the manuscript. Revised portion are marked in red in the paper.

The main corrections in the paper and the responds to the comments are as follows:

Reviewer #2: The emphasis of "improvement of sensitivity is significant" in the Ablation is not proper. First, the 1% improvement cannot be claimed as a significant one. Second, the comparison of sensitivity only is not fair since the specificity is worse. So I would suggest a rephrase. Apart from this, my other comments are addressed.

**Response:** Thanks for the suggestion. We think this comment is very reasonable and we have rephrased the sentence. In fact, the 1% improvement is slightly, so in order not to confuse the reader, we directly replaced the percentage of increase with values. And in manuscript we rephrase the sentence as:“When we added our proposed SA module, we can see that all indicators except for the specificity have increased, among which MCC, SE, ACC, AUC and F1 scores increase to 0.8055, 0.8252, 0.9569, 0.9822, and 0.8289, respectively. These results demonstrate the effectiveness of the proposed scale-attention module to a certain extent.”
